# Antibiofilm assay for antimicrobial peptides combating the sulfate‐reducing bacteria *Desulfovibrio vulgaris*

**DOI:** 10.1002/mbo3.1376

**Published:** 2023-08-21

**Authors:** Lena Stillger, Lucile Viau, Dirk Holtmann, Daniela Müller

**Affiliations:** ^1^ Institute of Bioprocess Engineering and Pharmaceutical Technology University of Applied Sciences Mittelhessen Giessen Germany; ^2^ Institute of Process Engineering in Life Sciences Karlsruhe Institute of Technology Karlsruhe Germany; ^3^ Institute of Pharmaceutical Technology and Biopharmacy Philipps‐University Marburg Marburg Germany

**Keywords:** antimicrobial peptides, biocorrosion, biofilm, biofilm monitoring, *Desulfovibrio vulgaris*

## Abstract

In medical, environmental, and industrial processes, the accumulation of bacteria in biofilms can disrupt many processes. Antimicrobial peptides (AMPs) are receiving increasing attention in the development of new substances to avoid or reduce biofilm formation. There is a lack of parallel testing of the effect against biofilms in this area, as well as in the testing of other antibiofilm agents. In this paper, a high‐throughput screening was developed for the analysis of the antibiofilm activity of AMPs, differentiated into inhibition and removal of a biofilm. The sulfate‐reducing bacterium *Desulfovibrio vulgaris* was used as a model organism. *D. vulgaris* represents an undesirable bacterium, which is considered one of the major triggers of microbiologically influenced corrosion. The application of a 96‐well plate and steel rivets as a growth surface realizes real‐life conditions and at the same time establishes a flexible, simple, fast, and cost‐effective assay. All peptides tested in this study demonstrated antibiofilm activity, although these peptides should be individually selected depending on the addressed aim. For biofilm inhibition, the peptide DASamP1 is the most suitable, with a sustained effect for up to 21 days. The preferred peptides for biofilm removal are S6L3‐33, in regard to bacteria reduction, and Bactenecin, regarding total biomass reduction.

## Graphical abstract

This study aimed to develop a fast and realistic antibiofilm assay for screening antimicrobial peptides (AMPs) for biocorrosive bacteria. Effective AMPs for both inhibition and removal of the biofilm could be identified and long‐term inhibition of biofilm was possible for up to 21 days.

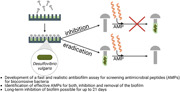

## 1 INTRODUCTION

The majority of microorganisms live as aggregates forming a biofilm (Flemming et al., 2002). Thereby, biofouling describes the phenomenon of undesirable biofilms (Flemming, 2002). Here, it can lead to numerous disruptions of processes, either in the medical field and human health due to the colonization of implants (usually titanium) or medical devices (usually stainless steel) through numerous multi‐resistant bacteria like ESKAPE pathogens (*Enterococcus faecium*, *Staphylococcus aureus*, *Klebsiella pneumoniae*, *Acinetobacter baumannii*, *Pseudomonas aeruginosa*, and *Enterobacter* species) and their associated potential for infection (Vetrivel et al., 2021), or in industrial plants such as water circuits or the oil and gas industry (Flemming, 2002). In industrial processes, biocorrosion bacteria have an important role in attacking metals through various mechanisms (Chugh et al., 2020). In this context, sulfate‐reducing bacteria (SRB) and sulfur‐oxidizing bacteria (SOB) should be mentioned in particular, as they can cause immense corrosion damage by forming a sulfur cycle (Chugh et al., 2020).

The problem in combating biofilms is that in contrast to the planktonic mode, sessile cells are better protected through their production of extracellular polymeric substances (EPS), which act as a diffusion barrier against extrinsic substances (Dunsing et al., 2019). Additionally, the so‐called persister cells, which are characterized by resting cell metabolism, are immune to antibiotics (Wood, 2016). These problems result in a higher required biocide concentration compared to combating suspension bacteria (Saleh et al., 2021). Due to high resistance and high cytotoxicity (Alhajjar et al., 2022; McLaughlin et al., 2021), the application of biocides is limited, and the development of new antimicrobial and antibiofilm candidates is crucial. In recent years, antimicrobial peptides (AMPs) have emerged as a new treatment strategy (Luo et al., 2023). Thereby, AMPs can lead to cell death via electrostatic interaction with the bacterial membrane and subsequent incorporation into the membrane and formation of pores (membranolytic mode of action) (Zhang et al., 2021). In addition, AMPs can inhibit intracellular metabolic pathways (Zhang et al., 2021). In addition to killing cells directly, AMPs can act on any state of biofilm formation, including regulation of the cell signaling pathway by the AMP LL‐37 (Overhage et al., 2008) or reduction of EPS by targeting a major polysaccharide of EPS matrix by the AMP hepcidin 20 (Brancatisano et al., 2014). Although there are numerous modes of action of AMPs against biofilms, up to this time, only the effectiveness against the planktonic cells of biocorrosion bacteria has been shown (Stillger et al., 2023).

Analysis of the complete biofilm is not possible due to its complex composition. Nevertheless, various analytical methods (colorimetric, microscopic, etc.) and different assays (BioFilm Ring Test, flow chamber, etc.) are available to characterize the different biofilm aspects (Azeredo et al., 2017). A limitation of these methods is often insufficient throughput, which is important for the development of new compounds. To overcome this, the Calgary Biofilm Device, a 96‐well biofilm assay, has been developed. Ceri et al. (1999). The optimization of this assay, with the use of microcentrifuge tubes as growth surfaces in the respective wells, ensures flexibility to the respective conditions, as well as high throughput at the same time. Additionally, this method is more economical than the Calgary Biofilm Device and can be implemented with common laboratory equipment (Reiferth et al., 2022). It can be used to determine the minimum biofilm inhibitory concentration (MBIC), as well as the minimum biofilm eradication concentration (MBEC). Therefore, the biofilm is stained by crystal violet, and in the case of MBEC, viable microorganisms are measured through a recovery plate.

This study aims to establish this assay to determine the MBIC and MBEC on biocorrosive bacteria and quantify the activity of AMPs in a high‐throughput method. For the first time, the application of AMPs against a representative of SRB, *Desulfovibrio vulgaris*, for inhibition and eradication of biofilm is presented. The utilization of steel rivets as growth surface ensures realistic material conditions.

## 2 MATERIALS AND METHODS

### 2.1 Synthesis of peptides

The peptides were synthesized with 9‐fluorenylmethoxycarbonyl chemistry using the microwave‐assisted peptide synthesizer LibertyBlue^TM^ (CEM GmbH) in 0.1 mM scale. Peptides were checked for purity (reversed‐phase high‐performance liquid chromatography) and identity (liquid chromatography‐mass spectrometry). For details regarding the above procedures see Stillger et al. (2023). The peptides with associated sequences used in this study are listed in Table 1.

**Table 1:** Collection of antimicrobial peptides used in this study with their corresponding sequence.

Peptide name	Sequence
L5K5W‐W4I6	KKLWKWLKKLL
S6L3‐33	FKKFWKWFRRF
Bactenecin	RLCRIVVIRVCR
DASamP1	FFGKVLKLIRKIF

### 2.2 Stains and cultivation

*D. vulgaris* DSM 644 was cultivated in Postgate medium (DSMZ medium 63) with a modification of 0.004 g/L FeSO_4_ × 7H_2_O and 0.3 g/L trisodium citrate. The medium was flushed with nitrogen gas. Incubation was performed at 37°C without shaking.

### 2.3 MBIC assay

**Figure A2:**
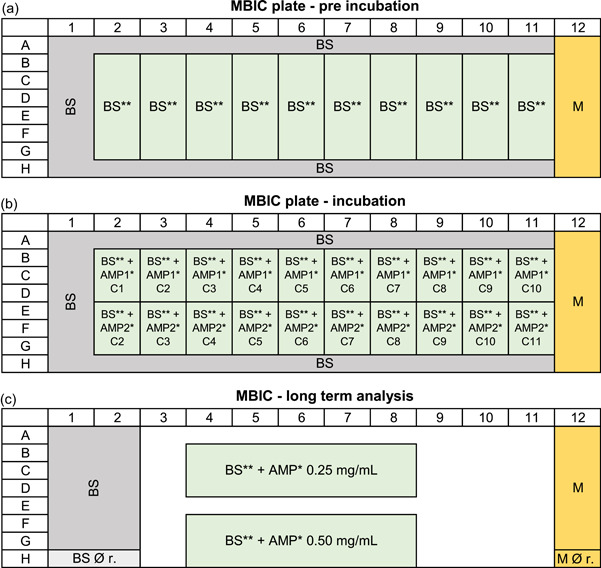
Pipetting scheme of each plate for minimum biofilm inhibition concentration (MBIC) preincubation (a), incubation (b), and long‐term analysis (c). AMP, antimicrobial peptide; BS, bacterial suspension; C, concentration of AMP solution, 120 µL volume per well; M, medium; r., rivets. *20 µL volume per well; **20 µL per well.

Steel rivets DIN660 4 × 15 mm (Würth‐Gruppe) served as the growth surface. These were applied in the following pretreated stages: (1) mechanically untreated; (2) roughened: two turns with silicon carbide sandpaper with a grit size of 180; (3) polished: two turns with silicon carbide sandpaper with a sequential grit size of 320, 400, 600, 800, and 1200. The steel rivets were then cleaned in acetone, 2‐propanol, and ethanol (Carl Roth GmbH & Co. KG) for 5 min each using an ultrasonic bath, frequency 47 KHz (Branson Ultrasonic Corporation). The process was repeated a second time before sterilization using a UV lamp for 1 h. After 30 min, the rivets were rotated to ensure uniform exposure. The bacterial culture was subcultured to a starting OD600 of 0.007. A measure of 120 µL of bacterial culture per well was added as growth check (*n* = 14), 100 µL bacterial culture per well for AMP test (*n* = 3 per AMP concentration), and 120 µL medium per well as sterile control (*n* = 4) to a 96‐microtiter plate (polystyrene). Subsequently, each well was fitted with a rivet. Preincubation for 6 h was done. Afterward, 20 µL of AMP solution (sequential twofold dilution of 1 mg/mL to 1.95 µg/mL) was added to the corresponding well (in Figure A2, a pipetting scheme is shown). After a further incubation time of 66 h, biomass in the solution (OD600) and the biofilm formation were characterized by crystal violet staining. For crystal violet staining, the rivets were removed and washed with 0.9% NaCl. Subsequently, the biofilm was fixed using 99% methanol (incubation 5 min) and dried for 60 min. Subsequently, staining was performed using 0.5% crystal violet (incubation 30 min), washing 3× with water, and drying for 120 min. Destaining was performed with ethanol/acetone (80/20) for 20 min and detection at 570 nm. The respective steps were realized by transferring the rivets into a well plate with the appropriate solution (140 µL/well). Except for the crystal violet staining, the assay was carried out in an anaerobic chamber (Coy Laboratory Products Inc.). Anaerobic cultivation was realized through air‐tight containers including an Oxoid anaerobic bag (Thermo Fisher Scientific Inc.) to ensure anaerobic conditions. Routine measurements of OD600 and OD570 were performed using the InfiniteM200Pro plate reader (Tecan Group AG). For measurements in the anaerobic chamber, OD600 was measured with a Stratus microplate reader (Cerillo Inc.). This enables the determination of long‐term MBIC effects.

### 2.4 MBEC assay

**Figure 1:**
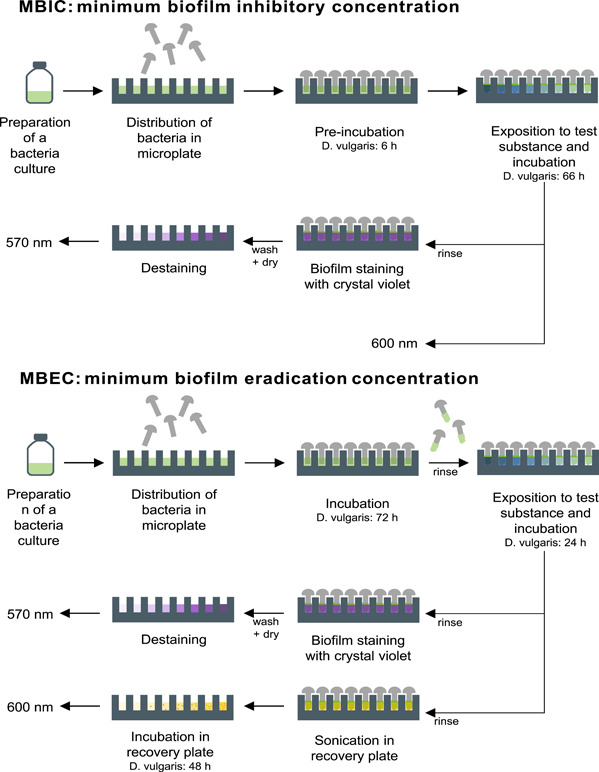
Overview of the individual steps of MBIC and MBEC assay for *Desulfovibrio vulgaris*.

**Figure A3:**
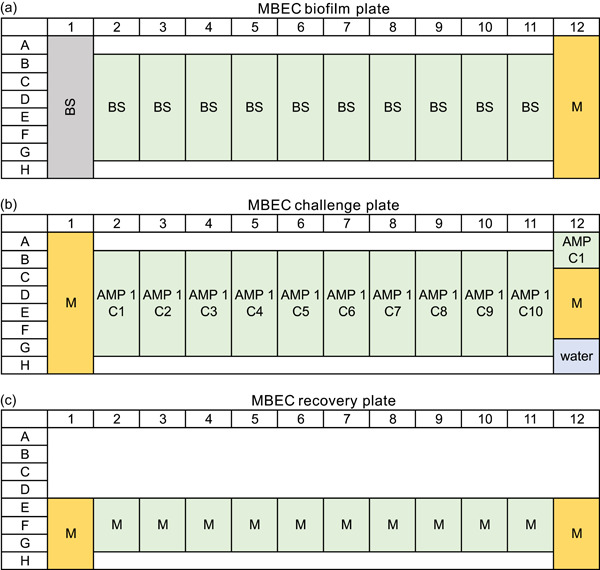
Pipetting scheme of each plate for minimum biofilm eradication concentration (MBEC) biofilm plate (a), challenge plate (b), and recovery plate (c). AMP, antimicrobial peptide; BS, bacterial suspension; C, concentration of AMP solution, 120 µL volume per well; M, medium.

The preparation of the MBEC assay was similar to the MBIC. A measure of 120 µL of bacterial suspension was added per well as growth check (*n* = 8) or as AMP test (*n* = 6 per AMP concentration) and 120 µL of medium as sterile control (*n* = 8). The microtiter plate was incubated for 72 h. Afterward, the rivets were transferred to the challenge plate consisting of 120 µL of AMP solution (1 mg/mL to 1.95 µg/mL), 120 µL of medium for growth check (*n* = 8), 120 µL of medium (*n* = 4) or 120 µL of water (*n* = 2) or 120 µL of AMP with a concentration of 1 mg/mL (*n* = 2) for sterile control (in Figure A3, a pipetting scheme is shown). Incubation was performed for 24 h. The rivets were then washed with 0.9% NaCl and half of them were stained with crystal violet (see MBIC). The other half was transferred to the recovery plate containing 120 µL of medium per well and placed in the ultrasonic bath for 30 min. The rivets were removed and the microtiter plate was incubated for 48 h. After the incubation period of 48 h, OD600 was measured. An overview of the individual steps of the MBIC/MBEC assay is shown in Figure 1.

## 3 RESULTS

### 3.1 Adaptation of the growth surface for *D. vulgaris*

**Figure A4:**
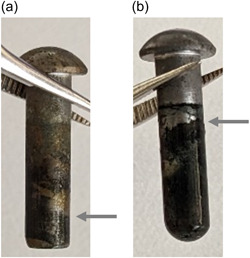
Mechanically untreated (a) and polished (b) rivets with *Desulfovibrio vulgaris* biofilm after 72 h incubation, gray lines indicate the height of the biofilm.

Based on Table 2, the OD570 of biofilms on PCR tubes indicated a low concentration of biomass with a high standard deviation of 50%. When steel rivets were used, the OD570 increased significantly. Additionally, OD570 increased with increasing pretreatment—OD570 of the polished rivets (3) higher than roughened rivets (2) higher than mechanically untreated rivets (1). The polished steel rivets showed the highest concentration of biofilm with an OD570 of 1.30 simultaneously with a low variation. For the polished rivets, a constant biofilm over the entire contact surface between the rivet and the bacterial suspension was observed. This was in distinct contrast to the untreated rivets, where the biofilm was mainly confined to the edge (see Figure A4).

**Table 2:** Biofilm of *Desulfovibrio vulgaris* after 72 h on different growth surfaces: PCR tubes; rivets: mechanically untreated; roughened: silicon carbide sandpaper (grit size: 180); polished: silicon carbide sandpaper (grit size: 320, 400, 600, 800, and 1200); staining by crystal violet and measurement OD570 nm; mean and standard deviation were determined by error propagation.

	PCR tubes	Steel rivets		
		Mechanically untreated	Roughened	Polished
OD570 nm	0.24 ± 0.12	0.59 ± 0.23	0.95 ± 0.14	1.30 ± 0.21

### 3.2 Biofilm inhibition through AMPs

**Figure 2:**
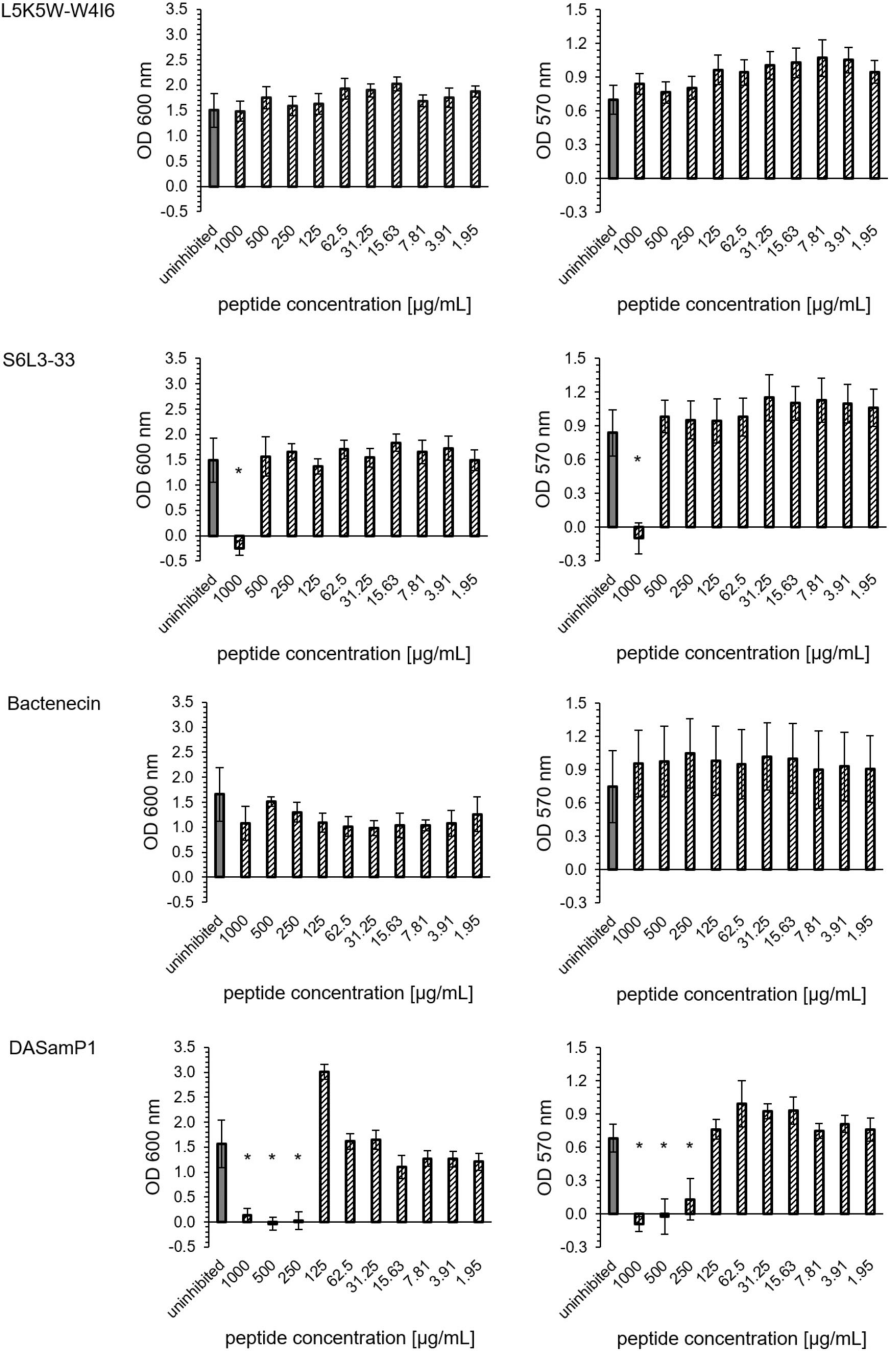
Minimum biofilm inhibitory concentration (MBIC) assay for *Desulfovibrio vulgaris*: OD600 and OD570 of the challenge plate (72 h incubation) for different concentrations of four antimicrobial peptides: L5K5W‐W4I6, S6L3‐33, Bactenecin, DASamP1 (black striped), and uninhibited sample (gray); mean and standard deviation were determined by propagation of error; asterisks indicate significant reduction (*p* = 0.005).

The peptides L5K5W‐W4I6 and Bactenecin showed no biofilm inhibitory effects. The peptide S6L3‐33 had a MBIC95% of 1000 µg/mL. The peptide DASamP1 had the lowest minimum biofilm inhibitory concentration with a MBIC95% of 250 µg/mL (Figure 2). These results were consistent for both OD600 and OD570.

### 3.3 Long‐term effectiveness of biofilm inhibition through AMPs

**Figure 3:**
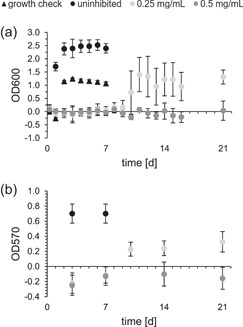
Long‐term analysis for the peptide DASamP1 against *Desulfovibrio vulgaris*: OD600 (a) and OD570 (b) over time (days) for uninhibited bacteria culture (black circle), growth check without rivets (black triangles), and inhibited bacteria culture with a peptide concentration of 0.25 mg/mL (light gray) and 0.5 mg/mL (dark gray); mean and standard deviation were determined by the propagation of error.

DASamP1 was selected for long‐term study based on its best activity in the previous MBIC assay. For both the uninhibited samples (black circles) and the growth check without rivet (black triangles), an increase in OD600 was initially detected within the first 2 days, remaining constant thereafter. At a peptide concentration of 250 µg/mL (light gray), no bacterial growth could be detected up to Day 9. Subsequently, an increase in OD600 was detectable and, from Day 11 onwards, remained almost constant until the end of the study (Figure 3a). While no positive OD570 was detectable until Day 7, an increase in the OD570 was detected on Day 10, which remained almost constant over the further period (Figure 3b). It should be mentioned that both OD600 and OD570 of the inhibited samples did not reach the identical values of the uninhibited sample during the investigated period. Instead, they reached approximately half the values of the uninhibited sample. In contrast, no increase in OD600 could be detected over the complete period investigated at a peptide concentration of 500 µg/mL (dark gray), see Figure 3a. This was also visible in the crystal violet staining, where no increase in OD570 could be detected (Figure 3b).

### 3.4 Peptide stability over 21 days

**Figure 4:**
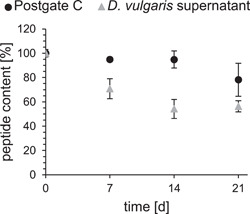
Long‐term stability for the peptide DASamP1 in Postgate C medium (black) and in the supernatant of a 7‐day‐old culture of *Desulfovibrio vulgaris* (gray), analyzed with reversed‐phase high‐performance liquid chromatography; 100% peptide content corresponds with the peptide content at 0 h. Values are shown as means with standard deviation, *n* = 3.

Peptide in the medium (Figure 4, black) was almost stable until Day 14. Over time, however, there was a slight decrease of up to slightly less than 80%. For the measurements in the culture supernatant (Figure 4, gray), only a peptide content of 70% could be detected after 7 days. The decrease went on in the following days, resulting in a peptide content of only 56% after 21 days.

### 3.5 Biofilm eradication through AMPs

**Figure 5:**
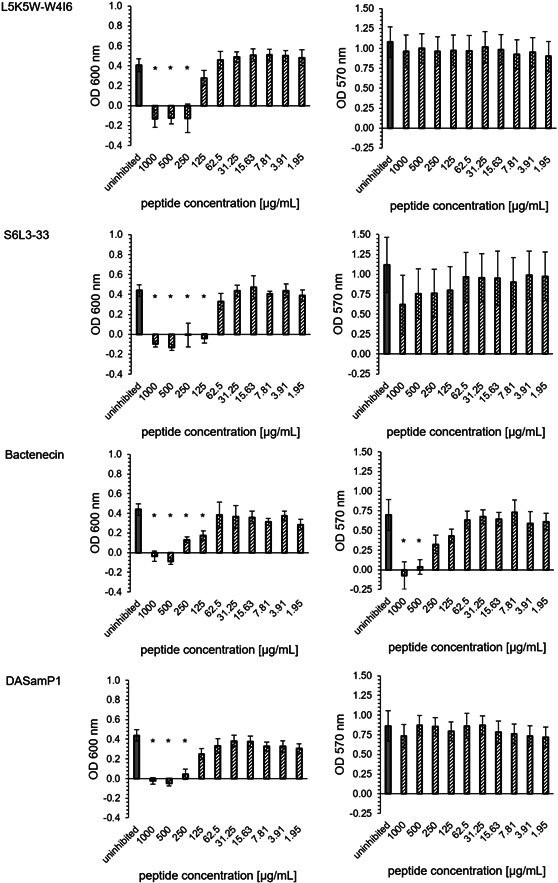
Minimum biofilm eradication concentration (MBEC) assay for *Desulfovibrio vulgaris*: OD600 of the recovery plate (48 h incubation) and OD570 of the challenge plate (72 h incubation) for different concentrations of four antimicrobial peptides: L5K5W‐W4I6, S6L3‐33, Bactenecin, DASamP1 (black striped), and uninhibited sample (gray); mean and standard deviation were determined by error propagation; asterisk indicates significant reduction (*p* = 0.005).

S6L3‐33 was detected as the most effective peptide in regard to OD600 with a MBEC95% of 125 µg/mL. L5K5W‐W4I6 and DASamP1 reached each a MBEC95% of 250 µg/mL. Bactenecin exhibited a 95% reduction of OD600 up to 500 µg/mL and a partial effect could be detected at two subsequent concentration levels (250 and 125 µg/mL). For the removal of the total biofilm mass (OD570), only the peptide Bactenecin was able to contribute. The concentration curve is comparable to the measurement at OD600. Up to a peptide concentration of 500 µg/mL, complete removal of the biofilm was observed. For the concentrations 250 and 125 µg/mL, a partial removal could be detected (Figure 5).

## 4 DISCUSSION

For the establishment of the Calgary assay modified by Reiferth et al. (2022) for the SRB *D. vulgaris*, all growth‐critical steps were performed in an anaerobic chamber, and only the crystal violet staining was performed under aerobic conditions. All required fluids were flushed with N_2_. The selection of growth medium and temperature was based on the optimal conditions for the planktonic growth of *D. vulgaris*. In naturally occurring biocorrosion, such as in water cycles, the nutrient supply is very different from the medium used here, and the temperature could reach higher or lower values. The analysis under realistic conditions may be considered in further experiments. In addition to transferring the assay to anaerobic conditions, the incubation periods of *D. vulgaris* were adjusted. This adjustment was oriented to the growth curve of Zhang et al. (2016), where the mean exponential phase of sessile growth was reached after an incubation period of 72 h. A modification of the material's adhesion surface is necessary due to very low and heterogeneous biofilm formation on previously used polypropylene tubes (see Table 2). A large number of different materials have been described in the literature for the colonization of SRB biofilms, which are mostly based on metals such as steel, titanium, and aluminum (Rao & Feser, 2023; Ru Jia et al., 2017). For further assay development, steel rivets were used. This offers realistic material conditions for the usage of the corrosive SRB. The mechanical pretreatment of the rivets was based on de Andrade et al. (2020). Here, the increase of the surface area by treatment with silicon carbide sandpaper leads to an increased roughness, which would explain the improved biofilm accumulation. Comparable studies were also performed with mechanically pretreated metals (Rao & Feser, 2023; Ru Jia et al., 2017). By utilizing rivets produced by other metals, such as aluminum, it is also possible to evaluate biocorrosion effects on other metals with minimal effort, as well as to investigate other issues, such as the analysis of bacteria relevant to medical technology and human health, through the use of stainless steel or titanium rivets. Especially in the clinical field, biofilms caused by ESKAPE pathogens and the associated problem of limited antibiotics are an increasing problem (De Oliveira et al., 2020; Nasser et al., 2020). For example, *P. aeruginosa* can form biofilms on a variety of materials, including stainless steel, posing a significant risk to many medical devices (Guilbaud et al., 2017). Biofilm formation also frequently occurs on titanium, a commonly used implant material, resulting in inflammation and implant failure (Sarfraz et al., 2022; Souza et al., 2021). By simply modifying the rivet material, screening of new antibiofilm substances can be carried out under realistic conditions, expanding the range of applications beyond biocorrosion. In addition to this flexible, simple, and fast assay, the cost‐efficiency and adaptability to different realistic conditions through the usage of rivets made of different metals could be mentioned as further advantages of this modified MBIC/MBEC assay. Nevertheless, each biofilm assay, as well as each analysis method, has advantages and disadvantages and should be selected according to the respective aim (Azeredo et al., 2017). The presented assay here, with its corresponding analytics based on OD600 measurement and crystal violet staining, was primarily designed for high‐throughput screening. The established method of crystal violet staining exhibits some limitations: Toxicity, end‐point measurements, nonspecific binding to negatively charged molecules, and therefore no differentiation between cells and biofilm components, and low reproducibility (Amador et al., 2021; Azeredo et al., 2017). However, this method provides a simple indication of the general biofilm activity of substances and is therefore well suited for screening purposes. For detailed investigations of the mechanisms of action of the substances, other methods should be used afterward (Azeredo et al., 2017). The peptides that were tested here exhibited good effectiveness (minimal inhibitory concentration <125 µg/mL) against planktonic *D. vulgaris* (Stillger et al., 2023 *)* and were therefore used for further studies against SRB biofilm presented in this paper. The optimized form L5K5W‐W4I6 was used instead of the original peptide L5K5W, although the lipidated and amidated optimization was avoided due to better comparability of the individual four peptides.

The OD600 measurement in the MBIC assay is influenced due the formation of black iron sulfide by the SRBs. For this purpose, following the method by Bernardez & de Andrade Lima (2015) and the transfer to the 96‐well plate format according to Wood et al. (2019), the iron precipitates can be dissolved by hydrochloric acid. This is not necessary in this case, since the significance of the OD600 is not affected. The peptide DASamP1 is the most effective against biofilm inhibition (MBIC95 of 250 µg/mL) and is only slightly lower compared to its planktonic effectiveness against *D. vulgaris*. In further studies, this peptide was also able to inhibit an early biofilm stage of multiresistant *Staphylococcus aureus* (Menousek et al., 2012) and consequently has great potential as a short and “simple” peptide for inhibiting biofilms. The peptide structure of DASamP1 suggests a membranolytic mode of action (Menousek et al., 2012). However, the exact mode of action, in particular the effective inhibition of a biofilm, is not yet known. A key factor in preventing biofilm formation by corrosive bacteria is to maintain the effect of the peptides for as long as possible. Due to the good effectiveness of DASamP1, this peptide was selected for long‐term observation. Both MBIC (250 µg/mL) and double MBIC (500 µg/mL) were used as peptide concentrations. From Day 10 onwards, bacterial growth and biofilm formation could be detected using the MBIC. This is due to the decreasing stability of the peptide in the bacterial supernatant (see Figure 4), where the peptide concentration was halved at this time. The available concentration is thus only 1/2 MBIC, which was found to be too low to inhibit the biofilm in the previous experiment. It was not observed that the identical OD600 and OD570 were obtained in the inhibited samples as in the uninhibited samples. Instead, the values were about one‐half lower, which could be due to the partial efficacy of the AMPs still present. In contrast, the doubled concentration (500 µg/mL) was effective over the entire period of 21 days. Halving the peptide concentration over time resulted in a concentration of around 250 µg/mL, which is equivalent to MBIC and thus still effective. To achieve the longest possible biofilm inhibition, it is necessary to either apply a higher concentration than the real MBIC to buffer peptide degradation or to avoid peptide degradation by using d‐amino acids, nonnatural amino acids, or cyclization while maintaining peptide activity (Gentilucci et al., 2010; Molhoek et al., 2011). However, a longer observation was not further possible with this setup due to starting evaporations. Instead, the long‐term study should be performed in a closed system. Hereby, it was possible to show the principal biofilm inhibition of different peptides after a certain period (72 h) as well as their long‐term efficacy, while applying the same materials. Therefore, different aims can be targeted with this assay material. The other three tested peptides showed a lower biofilm inhibitory activity. In the case of S6L3‐33, its amphiphilic structure suggests a membranolytic mode of action (He et al., 2007). The same is true for L5K5W, so it is assumed that the modified version L5K5W‐W4I6 acts identically to the original peptide (Kang et al., 2009; Kim et al., 2013). Bactenecin, which is linear due to reducing conditions of the medium, also has a membranolytic effect, and barrel stave is assumed in this context (Wu & Hancock, 1999). All tested peptides showed a significant loss of activity regarding biofilm inhibition compared to their effectiveness against planktonic cells. This can be explained by the time lag of peptide addition in the MBIC assay compared to MIC determination and the associated challenges of biofilm structure. In particular, the EPS matrix acts as a diffusion barrier, and therefore higher concentrations are often necessary to combat biofilms (Dunsing et al., 2019; Saleh et al., 2021). Why the peptide DASamP1 is best able to overcome these challenges is currently unexplored and more detailed analyses of biofilm mechanism are needed.

In the MBEC assay, all four peptides showed killing activity against the bacteria, based on the OD600 measurement of the recovery plate. However, as this is only a turbidity measurement, where bacterial fragments can also be considered, this method is flawed. A more accurate method is the determination of cell number, which is a more complex method and therefore not considered for high throughput. For the reduction of the total biomass, only the peptide Bactenecin was successful. The remarkably strong antibiofilm activity of Bactenecin, specifically in eradicating total biofilm mass, may be related to its structure. Bactenecin has a cyclic form with a hydrophobic ring and a hydrophilic tail in an oxidative milieu due to the presence of two cysteines (Yari et al., 2019). This characteristic structure enables surfactant‐like interactions. Thereby, surface‐active peptides promote the degradation and detachment of biofilms by reducing surface tension (Banaschewski et al., 2015; Mandal et al., 2013). Especially, the MBEC assay indicates that different substances have different points of interaction. For efficient removal of the biofilm, it is necessary to develop a mixture of different peptides, each with different biofilm target points.

In this study, the successful application of AMPs, both to inhibit a biofilm and to remove an existing biofilm, was shown against *D. vulgaris*, a representative of SRB, which is considered a major agent against biocorrosion. Therefore, a modified Calgary assay was implemented. This assay allows realistic conditions through the use of metal rivets and is flexible depending on the required conditions, allowing different bacteria (also anaerobic) and different growth materials to be used. Thus, this assay can be used to screen new biofilm‐active substances fast, simply, and cost‐efficiently. In addition to the determination of the MBIC and the MBEC, this assay can be used for further analyses such as long‐term studies, and thus enables multiple possibilities with a single setup.

## AUTHOR CONTRIBUTIONS

**Lena Stillger**: Conceptualization (lead); data curation (lead); investigation (lead); methodology (lead); project administration (equal); visualization (lead); writing—original draft (lead). **Lucile Viau**: Investigation (supporting); visualization (supporting); writing—review and editing (supporting). **Dirk Holtmann**: Conceptualization (supporting); funding acquisition (lead); methodology (supporting); project administration (equal); writing—review and editing (supporting). **Daniela Müller**: Conceptualization (supporting); funding acquisition (lead); methodology (supporting); project administration (equal); writing—review and editing (supporting).

## CONFLICT OF INTEREST STATEMENT

None declared.

## ETHICS STATEMENT

None required.

## Figures and Tables

**Figure A1:**
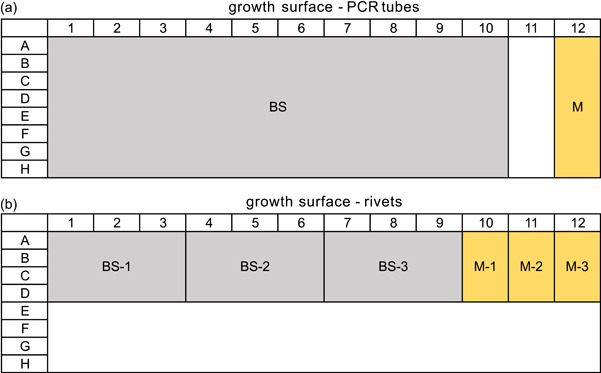
Pipetting scheme of each plate for biofilm growth on different growth surfaces: PCR tubes (a) and rivets (b): mechanically untreated (1), roughened (2), polished (3) for *Desulfovibrio vulgaris*. BS, bacterial suspension; M, medium.

## Data Availability

The data generated and analyzed during this study are included in this published article.

## APPENDIX A

Figure A1, Tables A1 and A2

**Table A1:** Two‐sample *t*‐test data (uninhibited and respective peptide concentration) for MBIC assay with *t* and *p* values; *⍺* was set at 0.005.

	*L5K5W‐W4I6*									
	OD600									
Peptide concentration (µg/mL)	1000	500	250	125	62.5	31.25	15.63	7.81	3.91	1.95
*t* Value	0.1063	−1.2232	−0.4172	−0.6148	−2.1136	−1.9817	−2.6267	−0.9206	−1.2086	−1.8858
*p* Value	0.9167	0.2401	0.6825	0.5479	0.0517	0.0661	0.0191	0.3718	0.2455	0.0788
*α*	0.0050	0.0050	0.0050	0.0050	0.0050	0.0050	0.0050	0.0050	0.0050	0.0050
	OD570									
Peptide concentration (µg/mL)	1000	500	250	125	62.5	31.25	15.63	7.81	3.91	1.95
*t* Value	−2.1005	−1.1943	−2.8865	−4.5451	−4.3521	−5.3642	−5.6880	−5.9926	−6.3474	−4.5323
*p* Value	0.0543	0.2509	0.0113	0.0004	0.0006	<0.0001	<0.0001	<0.0001	<0.0001	0.0004
*α*	0.0050	0.0050	0.0050	0.0050	0.0050	0.0050	0.0050	0.0050	0.0050	0.0050
	*S6L3‐33*									
	OD600									
Peptide concentration (µg/mL)	1000	500	250	125	62.5	31.25	15.63	7.81	3.91	1.95
*t* Value	5.6483	−0.2637	−0.6639	0.4896	−0.8351	−0.1970	−1.3631	−0.6395	−0.9267	0.0061
*p* Value	<0.0001	0.7956	0.5168	0.6315	0.4168	0.8465	0.1930	0.5322	0.3688	0.9952
*α*	0.0050	0.0050	0.0050	0.0050	0.0050	0.0050	0.0050	0.0050	0.0050	0.0050
	OD570									
Peptide concentration (µg/mL)	1000	500	250	125	62.5	31.25	15.63	7.81	3.91	1.95
*t* Value	8.4138	−1.5832	−1.2110	−1.1086	−1.4983	−3.1973	−2.8668	−2.9951	−2.7363	−2.3755
*p* Value	<0.0001	0.1342	0.2447	0.2851	0.1548	0.0060	0.0118	0.0091	0.0153	0.0313
*α*	0.0050	0.0050	0.0050	0.0050	0.0050	0.0050	0.0050	0.0050	0.0050	0.0050
	*Bactenecin*									
	OD600									
Peptide concentration (µg/mL)	1000	500	250	125	62.5	31.25	15.63	7.81	3.91	1.95
*t* Value	1.4092	0.4533	1.0879	1.7248	1.9662	2.0500	1.8833	1.8989	1.7341	1.1725
*p* Value	0.1806	0.6568	0.2938	0.1051	0.0681	0.0583	0.0792	0.0770	0.1034	0.2593
*α*	0.0050	0.0050	0.0050	0.0050	0.0050	0.0050	0.0050	0.0050	0.0050	0.0050
	OD570									
Peptide concentration (µg/mL)	1000	500	250	125	62.5	31.25	15.63	7.81	3.91	1.95
*t* Value	−2.6706	−2.7330	−3.7104	−2.8535	−2.4869	−3.4233	−3.0789	−1.6409	−2.2659	−2.0189
*p* Value	0.0175	0.0154	0.0021	0.0121	0.0252	0.0038	0.0076	0.1216	0.0387	0.0617
*α*	0.0050	0.0050	0.0050	0.0050	0.0050	0.0050	0.0050	0.0050	0.0050	0.0050
	*DASamP1*									
	OD600									
Peptide concentration (µg/mL)	1000	500	250	125	62.5	31.25	15.63	7.81	3.91	1.95
*t* Value	5.0564	5.6738	5.4041	−5.0934	−0.1825	−0.2742	1.6063	1.0380	1.0718	1.2595
*p* Value	0.0001	<0.0001	<0.0001	0.0001	0.8576	0.7877	0.1291	0.3157	0.3007	0.2271
*α*	0.0050	0.0050	0.0050	0.0050	0.0050	0.0050	0.0050	0.0050	0.0050	0.0050
	OD570									
Peptide concentration (µg/mL)	1000	500	250	125	62.5	31.25	15.63	7.81	3.91	1.95
*t* Value	12.1363	9.7672	7.3011	−1.2355	−3.9616	−3.8108	−3.6546	−0.9973	−2.0141	−1.1535
*p* Value	<0.0001	<0.0001	<0.0001	0.2356	0.0013	0.0017	0.0024	0.3344	0.0623	0.2668
*α*	0.0050	0.0050	0.0050	0.0050	0.0050	0.0050	0.0050	0.0050	0.0050	0.0050

**Table A2:** Two‐sample *t*‐test data (uninhibited and respective peptide concentration) for MBEC assay with *t* and *p* value; *⍺* was set at 0.005.

	*L5K5W‐W4I6*									
	OD600									
Peptide concentration (µg/mL)	1000	500	250	125	62.5	31.25	15.63	7.81	3.91	1.95
*t* Value	11.4992	15.1609	7.5388	2.8398	−1.0784	−2.5708	−2.8195	−2.9846	−2.9576	−1.7338
*p* Value	<0.0001	<0.0001	0.0007	0.0469	0.3301	0.0500	0.0371	0.0306	0.0316	0.1435
*α*	0.0050	0.0050	0.0050	0.0050	0.0050	0.0050	0.0050	0.0050	0.0050	0.0050
	OD570									
Peptide concentration (µg/mL)	1000	500	250	125	62.5	31.25	15.63	7.81	3.91	1.95
*t* Value	1.7963	2.0040	2.8670	2.1174	2.0647	1.1380	2.2305	4.0546	3.4008	4.3942
*p* Value	0.1324	0.1014	0.0351	0.0878	0.0939	0.3067	0.0761	0.0098	0.0192	0.0071
*α*	0.0050	0.0050	0.0050	0.0050	0.0050	0.0050	0.0050	0.0050	0.0050	0.0050
	*S6L3‐33*									
	OD600									
Peptide concentration (µg/mL)	1000	500	250	125	62.5	31.25	15.63	7.81	3.91	1.95
*t* Value	15.1769	15.7652	5.7289	9.4725	1.9264	0.0738	−0.4118	0.9291	0.0410	1.0891
*p* Value	<0.0001	<0.0001	0.0023	0.0007	0.1120	0.9441	0.6975	0.3955	0.9689	0.3258
*α*	0.0050	0.0050	0.0050	0.0050	0.0050	0.0050	0.0050	0.0050	0.0050	0.0050
	OD570									
Peptide concentration (µg/mL)	1000	500	250	125	62.5	31.25	15.63	7.81	3.91	1.95
*t* Value	2.4907	2.5471	2.7779	2.5530	1.1101	1.2467	0.9934	1.5631	1.0029	1.0521
*p* Value	0.0674	0.0635	0.0499	0.0631	0.3292	0.2805	0.3768	0.1931	0.3727	0.3521
*α*	0.0050	0.0050	0.0050	0.0050	0.0050	0.0050	0.0050	0.0050	0.0050	0.0050
	*Bactenecin*									
	OD600									
Peptide concentration (µg/mL)	1000	500	250	125	62.5	31.25	15.63	7.81	3.91	1.95
*t* Value	10.6620	14.4809	8.7056	6.1968	0.6865	1.0013	1.6624	3.4186	1.5013	3.4393
*p* Value	0.0001	<0.0001	0.0003	0.0016	0.5229	0.3627	0.1573	0.0189	0.1936	0.0185
*α*	0.0050	0.0050	0.0050	0.0050	0.0050	0.0050	0.0050	0.0050	0.0050	0.0050
	OD570									
Peptide concentration (µg/mL)	1000	500	250	125	62.5	31.25	15.63	7.81	3.91	1.95
*t* Value	5.1622	5.4968	2.9242	2.2446	0.5083	0.1992	0.4572	−0.2220	0.7712	0.6893
*p* Value	0.0036	0.0027	0.0329	0.0748	0.6329	0.8499	0.6667	0.8331	0.4754	0.5213
*α*	0.0050	0.0050	0.0050	0.0050	0.0050	0.0050	0.0050	0.0050	0.0050	0.0050
	*DASamP1*									
	OD600									
Peptide concentration (µg/mL)	1000	500	250	125	62.5	31.25	15.63	7.81	3.91	1.95
*t* Value	12.6770	13.8974	8.9943	4.1546	1.9783	1.2192	1.3740	2.7570	2.3984	3.1532
*p* Value	<0.0001	<0.0001	0.0003	0.0089	0.1048	0.2771	0.2278	0.0400	0.0617	0.0253
*α*	0.0050	0.0050	0.0050	0.0050	0.0050	0.0050	0.0050	0.0050	0.0050	0.0050
	OD570									
Peptide concentration (µg/mL)	1000	500	250	125	62.5	31.25	15.63	7.81	3.91	1.95
*t* Value	1.0639	−0.0673	0.0777	0.6137	−0.0036	−0.0902	0.6574	0.8885	1.1433	1.2767
*p* Value	0.3360	0.9489	0.9411	0.5663	0.9973	0.9317	0.5400	0.4149	0.3047	0.2578
*α*	0.0050	0.0050	0.0050	0.0050	0.0050	0.0050	0.0050	0.0050	0.0050	0.0050
